# The role of the neuroinflammation and stressors in premenstrual syndrome/premenstrual dysphoric disorder: a review

**DOI:** 10.3389/fendo.2025.1561848

**Published:** 2025-03-28

**Authors:** Ming Cheng, Zhaoshu Jiang, Jie Yang, Xu Sun, Nan Song, Chunyu Du, Zhenliang Luo, Zhen Zhang

**Affiliations:** ^1^ Yangsheng College of Traditional Chinese Medicine, Guizhou University of Traditional Chinese Medicine, Guiyang, Guizhou, China; ^2^ Research and Development Department, Qinhuangdao Shanhaiguan Pharmaceutical Co., Ltd, Qinhuangdao, Hebei, China; ^3^ Department of Integrated Traditional Chinese & Western Medicine, The Second Xiangya Hospital, Central South University, Changsha, Hunan, China

**Keywords:** neuroinflammation, premenstrual syndrome, premenstrual dysphoric disorder, stress, blood-brain barrier, kynurenine

## Abstract

Premenstrual syndrome (PMS) and premenstrual dysphoric disorder (PMDD) are prevalent emotional disorders in females, characterized by cyclic variations in physiological stress responses and emotional symptoms that correspond with the menstrual cycle. Despite extensive research, the underlying causes of these disorders remain elusive. This review delves into the neurobiological mechanisms connecting stress-induced neuroinflammation with PMS/PMDD. Additionally, it traces the conceptual development and historical context of PMS/PMDD. The review further evaluates clinical evidence on the association between PMS/PMDD and stress, along with findings from both clinical and animal studies that link these disorders to inflammatory processes. Additionally, the neurobiological pathways by which inflammatory responses may play a role in the pathogenesis of PMS/PMDD were elucidated, including their interactions with the hypothalamic-pituitary-ovary (HPO) axis, serotonin—kynurenine (5-HT—KYN) system, GABAergic system, brain-derived neurotrophic factor (BDNF), hypothalamic-pituitary-adrena(HPA)axis and. Future research is encouraged to further investigate the pathogenesis of PMS/PMDD through the perspective of neuroinflammatory responses.

## 1 Introduction

Premenstrual Syndrome (PMS) is a prevalent cyclic and recurrent condition affecting women of reproductive age, characterized by the manifestation of psychological and physical symptoms during the luteal phase of the menstrual cycle, which typically subside a few days after menstruation begins (1). Premenstrual Dysphoric Disorder (PMDD), Often considered a severe type of PMS, is recognized as a depressive disorder in the Diagnostic and Statistical Manual of Mental Disorders, Fifth Edition (DSM-5) (2). A systematic review and meta-analysis identified a global pooled prevalence of PMS at 47.8% (95% CI: 32.6–62.9) (3). The DSM-5 Text Revision (DSM-5-TR), a revised version of DSM-5 released by the American Psychological Association (APA) in 2022 (4), reports a prevalence rate of 1.3% for women whose symptoms meet the diagnostic criteria for PMDD, leading to functional impairment and occurring without co-existing mental disorders. The incidence rate for new cases over a follow-up period exceeding 40 months is 2.5% (95% confidence interval = 1.7–3.7%).

The first modern account of premenstrual symptoms was documented by Frank R.T. in 1931, who identified a condition he referred to as “premenstrual tension.” In 1953, Greene and Dalton introduced the term PMS. Since then, both the APA and ACOG have regularly updated the terminology, classification, and coding for PMS and PMDD. The historical progression of the names and classifications of PMS/PMDD is detailed in **Table 1**.

**Table 1:** Evolution of the names and disease classifications of PMS/PMDD.

Version	Name	Category	Code	Time	Author or Institution name
Frank	Premenstrual tension	/	/	1931	(5)
Greene R, Dalton K.	Premenstrual syndrome (PMS)	/	/	1953	(6)
DSM-III-R	Late luteal phase dysphoric disorder (LLPDD)	/	/	1987	(7)
DSM-IV	Premenstrual dysphoric disorder (PMDD)	Depressive Disorder Not Otherwise Specified	ICD-9-CM: 311	1994	(8)
ACOG	Premenstrual syndrome (PMS)	/	/	2000	(9)
DSM-IV-TR	Premenstrual dysphoric disorder (PMDD)	Depressive Disorder Not Otherwise Specified	ICD-9-CM: 311	2000	(10)
ACOG&ISPMD	Premenstrual syndrome (PMS)	/	/	2011	(11)
DSM-V	Premenstrual dysphoric disorder (PMDD)	Depressive disorders	ICD-10-CM: N94.3	2013	(2)
ICD-11	Premenstrual dysphoric disorder (PMDD)	Diseases of the genitourinary system	ICD-11: GA34.41	2019	
DSM-5-TR	Premenstrual dysphoric disorder (PMDD)	Depressive disorders	ICD-10-CM: F32.81	2022	(4)

As early as 1986, Kuczmierczyk and Adams in the U.S. began investigating the link between stress-induced physiological and emotional responses in patients with PMS (12). This focus on the effects of stress on PMS/PMDD has since gained significant attention, with researchers aiming to uncover the stress-related pathogenesis of these disorders. Evidence suggests that a substantial proportion of patients with PMS/PMDD have a history of trauma, violence, or abuse (13–16). Lee and Im’s review of 48 studies found that 92% confirmed the role of stress in the development of PMS/PMDD. They also identified both objective physiological stress responses and perceived stress as key predictors of the onset and severity of PMS/PMDD symptoms (14).Moreover, numerous studies have established a connection between PMS/PMDD symptoms—both physical and emotional—and alterations in inflammatory markers. For instance, interleukins, interferon gamma (IFN-γ), tumor necrosis factor alpha (TNF-α), hypersensitive C-reactive protein (hs-CRP), and Toll-like receptors (TLRs) have all been implicated in influencing PMS/PMDD symptoms to varying degrees (17–22). This paper delves into the relationship between stress and inflammatory responses, examining the connection between stress-induced inflammation and the neurobiological factors most pertinent to PMS/PMDD.

Stress refers to a state in which the body’s homeostasis is threatened or perceived to be threatened due to a large number of external or internal disruptive stimuli, known as “stressors” (23). When the body is exposed to stress stimuli, the stress system is activated, and regulation is achieved through the coordinated action of the hypothalamic-pituitary-adrenal (HPA) axis and the locus coeruleus (LC)/norepinephrine (NE)-autonomic nervous system (ANS). Activation of the HPA axis is accompanied by a decrease in the secretion of hypothalamic gonadotropin-releasing hormone (GnRH), which in turn leads to a reduction in luteinizing hormone (LH) and follicle-stimulating hormone (FSH) levels released by the anterior pituitary. FSH and LH together promote the synthesis and secretion of estrogen and progesterone (24).

A large body of research indicates that estrogen and progesterone can regulate inflammatory activity in the central nervous system (CNS), and the risk of PMS/PMDD is directly influenced by CNS inflammation (20). CNS inflammation can affect the regulation of the hypothalamic-pituitary-ovary axis (HPO), HPA axis (25), the serotonin (5-HT) system (26), the Gamma-aminobutyric acidergic (GABAergic) system (27) and brain-derived neurotrophic factor (BDNF) (7), all of which have long been associated with the risk of PMS/PMDD (20, 28–30).

The discussion explores key neurobiological mechanisms involved in neuroinflammation and PMS/PMDD, with a focus on the roles of the HPO, HPA, serotonin—kynurenine (5-HT—KYN) system, GABAergic system, BDNF.

## 2 Relationship between PMS/PMDD and stress: clinical evidence

### 2.1 Increased subjective perception of stress in patients with PMS

Early research suggests that women with severe PMS/PMDD perceive stressors more intensely and unpleasantly during the premenstrual phase compared to controls (31).In this study, we focus on stressors including hormonal stressors and physical stressors such as stress tests, etc. In addition, there is a correlation between depressive symptoms and increased perception of stressors in PMS patients (32). Gonda et al. (2010) found that women with pronounced PMS symptoms experienced greater premenstrual stress compared to postmenstrual stress when encountering identical life events, with a tendency to focus more on negative rather than positive experiences (31). A longitudinal study involving 93 medical students over 60 days demonstrated that increased work-related stress exacerbated luteal phase symptoms (33). Similarly, research in Spain revealed a strong association between high perceived stress levels and an increased likelihood of PMS (34). Lee and Im (2017) reported that Korean students studying in the U.S. experienced higher levels of stress and menstrual-related symptoms compared to domestic students, with perceived stress closely linked to the severity of premenstrual symptoms (14). In India, a study found a significant correlation between high stress levels (top quartile PSS scores) and the occurrence of premenstrual symptoms, including severe dysmenorrhea requiring medication (35). Negative attitudes towards menstruation have been shown to elevate subjective stress perception during the premenstrual phase, acting as a chronic stressor for patients with PMS (36). A German study suggested that increased premenstrual symptoms in women with PMS during the late luteal phase were linked to heightened daily rumination and perceived stress (37). Furthermore, a Jordanian study identified a statistically significant relationship between the severity of PMS and perceived stress scores (38). Hoyer et al. (2013) demonstrated that women with PMS exhibit an increasing trend in subjective stress measurements during the luteal phase (39). For the study of PMDD and stress, women with PMDD may exhibit heightened stress responses, particularly in high arousal negative affect (NA) states such as being upset or irritated (30). Subjective stress responses are primarily influenced by NA towards stressors (40). According to Russell’s circumplex model of affect, NA and positive affect (PA) can be further categorized into low arousal and high arousal states (41). Given that anger and irritability are key premenstrual symptoms (22, 42), distinguishing between arousal states is crucial when examining emotional responses in PMDD.

To sum up, women with PMS and PMDD experience heightened stress perceptions during the premenstrual phase, with negative affect and depression playing significant roles in amplifying this sensitivity. This heightened stress response is influenced by cultural factors, negative attitudes toward menstruation, and physiological stress markers.

### 2.2 Alternative coping strategies for stress regulation in women with PMS/PMDD

As previously discussed, patients with PMS/PMDD experience a range of physical and psychological changes during the premenstrual phase, often accompanied by heightened subjective stress perception in response to threatening stimuli (14). Research suggests that individuals with PMS/PMDD have been shown to exhibit a tendency toward avoidance or less effective coping strategies when managing stress, with an increased likelihood of adopting maladaptive coping mechanisms (43), potentially intensifying their premenstrual stress. It is hypothesized that patients with PMS/PMDD not only show impairments in daily emotional regulation strategies but are also more vulnerable to the negative impacts of maladaptive strategies, while struggling to benefit from adaptive strategies (44–46).

For PMS, these alternative coping strategies in stress response may be linked to they have differences or variations in neural function related to processing negative emotional information in patients with PMS. For example, patients with PMS display lower α asymmetry in the frontal lobes during resting states—a neurophysiological marker of emotional motivational reactivity—which may contribute to more intense negative emotional symptoms (47). Compared to healthy controls, patients with PMS exhibit increased gray matter (GM) volume in the precuneus/posterior cingulate cortex and thalamus, along with decreased GM volume in the insula. Receiver operating characteristic (ROC) analysis has highlighted differences in multiple brain regions, including the hippocampus, frontal lobe, and gyrus (48). In patients with PMS, increased functional connectivity is observed between the amygdala and specific regions of the frontal cortex, such as the medial prefrontal cortex (mPFC), anterior cingulate cortex (ACC), and right central gyrus, as well as with the right temporal pole and insula. Conversely, decreased functional connectivity is noted between the bilateral amygdalae and the right orbitofrontal cortex and right hippocampus. Notably, the strength of functional connectivity between the right amygdala and the right central gyrus, left ACC, and left mPFC is significantly positively correlated with Daily Record of Severity of Problems (DRSP) scores (49).

For PMDD, network analysis of brain function in patients with PMDD reveals changes in key metrics (e.g., characteristic path length, clustering coefficient, variability, local and global efficiency, centrality) during symptomatic and remission phases, indicating reduced functional network segregation and increased functional network integration. The difficulty in emotion regulation in PMDD is associated with the functional connectivity patterns of the striatum, thalamus, and prefrontal cortex, suggesting that neural dysfunction related to emotional information processing could contribute to stress response deficits (50).In PMDD patients, dorsomedial prefrontal cortex (dmPFC) activity is enhanced when anticipating negative emotional stimuli (51). During emotional regulation tasks, dorsolateral prefrontal cortex (dlPFC) activity is reduced in PMDD patients (52). Compared to healthy controls, women with PMDD exhibit heightened reactivity to social stimuli (compared to non-social stimuli) during the luteal phase, characterized by increased activation of the amygdala and insula and reduced activation of the anterior cingulate cortex. Notably, changes in progesterone levels in PMDD patients are positively correlated with alterations in amygdala reactivity (53).

In summary, studies have revealed physiological differences in brain activity within regions associated with emotional processing during the luteal phase between patients with PMS and non-PMS individuals (54).patients with PMS/PMDD experience heightened subjective stress perception, which is often exacerbated by maladaptive coping strategies and emotional regulation disorder. In particular, increased functional connectivity between the amygdala and frontal cortical regions is correlated with the severity of premenstrual symptoms, providing a potential neurobiological marker for emotional dysregulation in these conditions.

## 3 Association of PMS/PMDD with peripheral inflammation/central inflammatory response

### 3.1 From peripheral to central inflammatory responses

**Figure 1:**
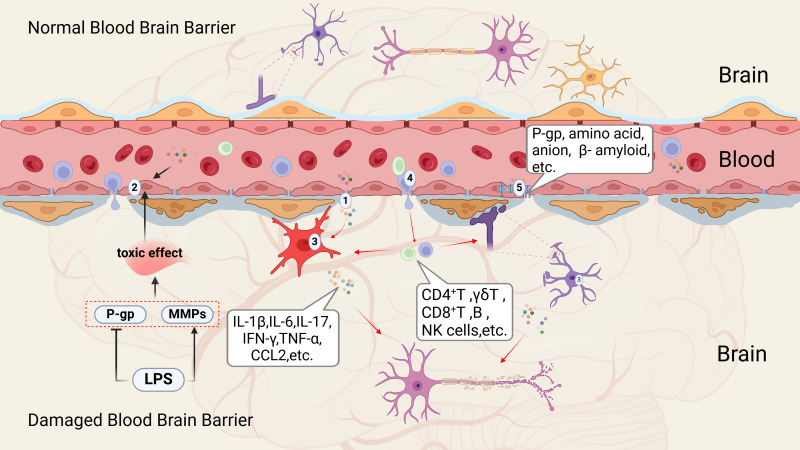
Mechanisms of peripheral inflammation transitioning to central inflammation (59). This process can be categorized into five critical aspects according to existing literature; (1) Alterations in tight junction; (2) Damage to cerebral vascular endothelial cells; (3) Activation of microglia and astrocytes; (4) Infiltration of peripheral immune cells; (5) Changes in various transport pathways and receptors. P-gp, P-glycoprotein (or P-glycoprotein 1); MMPs, matrix Metalloproteinases; LPS, lipopolysaccharide; IL-1β, interleukin-1 Beta; IL-6, interleukin-6; IL-17, interleukin-17; IFN-γ, interferon gamma; TNF-α, tumor necrosis factor alpha; CCL2, chemokine (C-C motif) ligand 2; CD4+T cells, CD4 T cell-mediated immunity; γδ T-cell, δ T Lymphocytes; CD8+T cells, Cytotoxic T lymphocytes; B cell, B lymphocytes; NK cell, natural killer cell.

When the body encounters stress, injury, illness, or infections from bacteria or viruses, the immune system is activated. Growing evidence indicates that psychological or emotional stress can similarly stimulate the peripheral immune system, resulting in the release of several pro-inflammatory cytokines (55). Following brain injury due to trauma, inflammation, infection, or disease, microglia and astrocytes within the brain become activated, leading to the production of various inflammatory mediators and chemokines, often accompanied by damage to the blood-brain barrier(BBB) (56). BBB plays a critical role in maintaining separation between the peripheral and central immune systems (57). Composed of endothelial cells connected by tight junctions, the BBB limits the entry of peripheral inflammatory cytokines, immune cells, metabolic byproducts, neurotoxic agents, and pathogens into the central nervous system (58). However, this barrier can be compromised during brain infections and neurodegenerative conditions, allowing peripheral immune cells to breach the BBB and initiate central inflammatory responses, which in turn can exacerbate BBB dysfunction. Recent study has identified five key mechanisms through which peripheral inflammation can lead to BBB disruption (See **Figure 1**) (60).

### 3.2 The relationship between PMS/PMDD and inflammatory responses

Preliminary research suggests a connection between PMS/PMDD and inflammatory responses. Although this area of research is still developing, it is significant that numerous studies have established a relationship between PMS/PMDD and stress, with stress being closely linked to inflammatory responses. Puder et al. investigated the relationship between inflammatory markers and both physical and psychological symptoms throughout the menstrual cycle in women of normal weight and those who are overweight. Their findings indicated that fluctuations in serum TNF-α and hs-CRP levels were associated with these symptoms across the menstrual cycle (18). Another study focused on young women revealed that those diagnosed with PMS had significantly higher mean levels of the interleukin (IL) family such as IL-4, IL-10, IL-12, and IFN-γ compared to healthy controls (17). In a review of the epidemiology and treatment of PMDD, Hantsoo et al. suggested that inflammatory factors might play a role in PMDD pathophysiology, with evidence showing elevated pro-inflammatory markers in women experiencing premenstrual symptoms compared to controls (22). Additionally, research by Bannister and Gold supports a possible connection between neuroinflammation and the underlying causes of PMDD (19, 20). A recent study in rats, examining the link between PMDD and inflammatory factors, this study used ovariectomy and hormone induction to prepare a PMDD model, found elevated expression levels of TLR-4, nuclear factor kappa beta (NF-κB) p65, TNF-α, IL-6, and IL-1β in the basal lateral amygdala in a model of PMDD compared to control rodents. Intervention with premenstrual comfort granules and fluoxetine reduced these inflammatory markers (21). Therefore, it is hypothesized that stress-induced inflammatory responses may be a key mechanism underlying the pathogenesis of PMS/PMDD.

Based on the above research, we speculate that the inflammatory response in PMS/PMDD involves both peripheral and central mechanisms. Stress, as a key factor in PMS/PMDD (46), can activate the peripheral immune system, inducing the release of pro-inflammatory cytokines, These cytokines can enter the brain and disrupt the BBB, allowing peripheral immune cells to infiltrate the central nervous system and trigger neuroinflammatory processes (56).

## 4 Potential biological mechanisms between PMS/PMDD pathogenesis and inflammatory response

### 4.1 HPO axis and neuroinflammation

#### 4.1.1 Sensitivity of sex hormone secretion to stress in PMS/PMDD

Research indicates a bidirectional relationship between the HPO axis, which governs the menstrual cycle, and the HPA axis, which regulates stress responses. These two functional axes not only overlap anatomically (61) but also interact in ways that influence each other’s physiological activities (62–64). The fluctuations in estrogen levels driven by the HPO axis before menstruation can modulate the activation and feedback mechanisms of the HPA axis, thereby affecting stress management (22, 65). Conversely, activation of the HPA axis under stress can influence the physiological functions of the HPO axis. This interaction is particularly relevant in patients with PMS, who exhibit a heightened neuroendocrine sensitivity to stress during the luteal phase (66, 67). Abnormalities in the HPA axis in patients with PMS may be closely linked to the interplay between the HPA and HPO axes.

Recent trends in neuroendocrinology have highlighted the connection between inflammatory processes and both psychological and physiological conditions, which share characteristics with premenstrual and postmenstrual syndromes (68). Estradiol and progesterone, known for their anti-inflammatory and antioxidant properties, decrease during the luteal phase, leading to increased oxidative stress in the endometrium. This oxidative stress elevates the production of pro-inflammatory prostaglandins, cytokines, chemokines, and matrix metalloproteinases (69). Certain chemokines may predict the severity of PMS symptoms, suggesting a potential interaction between the uterine-chemokine-brain axis (70). Additionally, elevated levels of pro-inflammatory cytokines have been observed in patients with PMDD (17). Furthermore, increased levels of C-reactive protein (CRP) have been positively correlated with the severity of PMDD symptoms, particularly in relation to mood, behavior, and pain (19). However, further research is necessary to fully understand the impact of inflammation, oxidative stress, and antioxidant status on the pathophysiology of PMDD (71).

#### 4.1.2 Neuroprotective mechanisms of sex hormones against neuroinflammation

Recent research has highlighted the significant role of estrogen receptors in microglial cells. 5-Androstene-3b,17b-diol (ADIOL), a metabolite of dehydroepiandrosterone (DHEA) produced by 17b-hydroxysteroid dehydrogenase type 14 (HSD17B14), has been shown to reduce inflammation in both microglia and astrocytes through an ERβ-dependent mechanism. Additionally, ADIOL engages with C-terminal binding protein (CtBP) in microglia *via* the ADIOL/ERβ/CtBP signaling pathway (72).Progesterone is well-known for its anti-inflammatory effects in both peripheral tissues and the central nervous system (73, 74). It modulates microglial function and counteracts estradiol’s influence on synaptic remodeling, a process mediated by progesterone receptors in rat microglia (75). Evidence suggests that various sex hormones confer neuroprotective and anti-neuroinflammatory effects through their interactions with microglia. *In vitro* studies using rat microglia have demonstrated that estradiol inhibits phagocytosis, reduces reactive oxygen species (ROS) production (76), and lowers the levels of pro-inflammatory molecules induced by LPS, including inducible nitric oxide synthase (iNOS), prostaglandin E2 (PGE2), and matrix metalloproteinase 9 (MMP-9) (77). The immunosuppressive effects of estradiol are mediated through estrogen receptors α (ERα) and β (ERβ), which regulate the expression of genes associated with neuroinflammation in rat microglia (78).

To sum up, in PMS/PMDD, the interaction between the HPO axis and the HPA axis contributes to heightened stress sensitivity, particularly during the luteal phase when fluctuations in estrogen and progesterone levels occur, this dysregulation exacerbates mood disturbances, irritability, and emotional instability (79). Additionally, the drop in these hormones during the luteal phase leads to increased inflammation and oxidative stress, which further amplify PMS/PMDD symptoms. Pro-inflammatory cytokines disrupt neuroprotective mechanisms, worsening the severity of symptoms (68).

### 4.2 5-HT-KYN as a potential mechanism of underlying PMS/PMDD

#### 4.2.1 Increased serotonin synthesis from tryptophan may alleviate PMS/PMDD symptoms

The involvement of serotonin in the pathogenesis of PMS/PMDD is most compellingly evidenced by studies utilizing drugs that modulate serotonin transmission in affected women. Serotonin-enhancing treatments, particularly selective serotonin reuptake inhibitors (SSRIs) that prevent serotonin reuptake in the synaptic cleft, have consistently demonstrated efficacy in mitigating PMS symptoms (80). Comparative studies on the efficacy of paroxetine, fluoxetine, escitalopram, sertraline, citalopram, and venlafaxine versus placebo have shown that SSRIs significantly alleviate overall self-reported symptoms, irrespective of whether administered continuously or during the luteal phase of the menstrual cycle (81). Additionally, some reports suggest that daily supplementation with 6 mg of tryptophan may aid in managing PMDD (82). Increased carbohydrate consumption prior to menstruation has also been associated with improvements in mood, carbohydrate cravings, and memory, likely due to enhanced brain tryptophan availability, thereby boosting 5-HT synthesis (83, 84). Moreover, evidence indicating that tryptophan deprivation induces premenstrual symptoms further implicates serotonin in the etiology of PMS/PMDD (29, 85).

#### 4.2.2 The 5-HT-KYN pathway is closely related to inflammation

5-HT is synthesized from the amino acid tryptophan *via* the enzyme tryptophan hydroxylase. However, an alternative metabolic route involves tryptophan’s conversion into KYN through the actions of indoleamine 2,3-dioxygenase (IDO) and tryptophan 2,3-dioxygenase (TDO) (86). In the kynurenine pathway, the initial step, catalyzed by IDO and TDO, converts tryptophan into kynurenine, which is subsequently metabolized into compounds that modulate glutamatergic neurotransmission *via* either the “excitotoxic” or “neuroprotective” pathways (87). In the excitotoxic pathway, 3-hydroxykynurenine (3-HK) is converted into quinolinic acid (QUIN) by kynurenine 3-monooxygenase (KMO), ultimately leading to nicotinamide adenine dinucleotide (NAD+) synthesis (88). Conversely, the neuroprotective pathway involves the conversion of kynurenine into kynurenic acid (KYNA) by kynurenine aminotransferase (KAT) (86).

**Figure 2:**
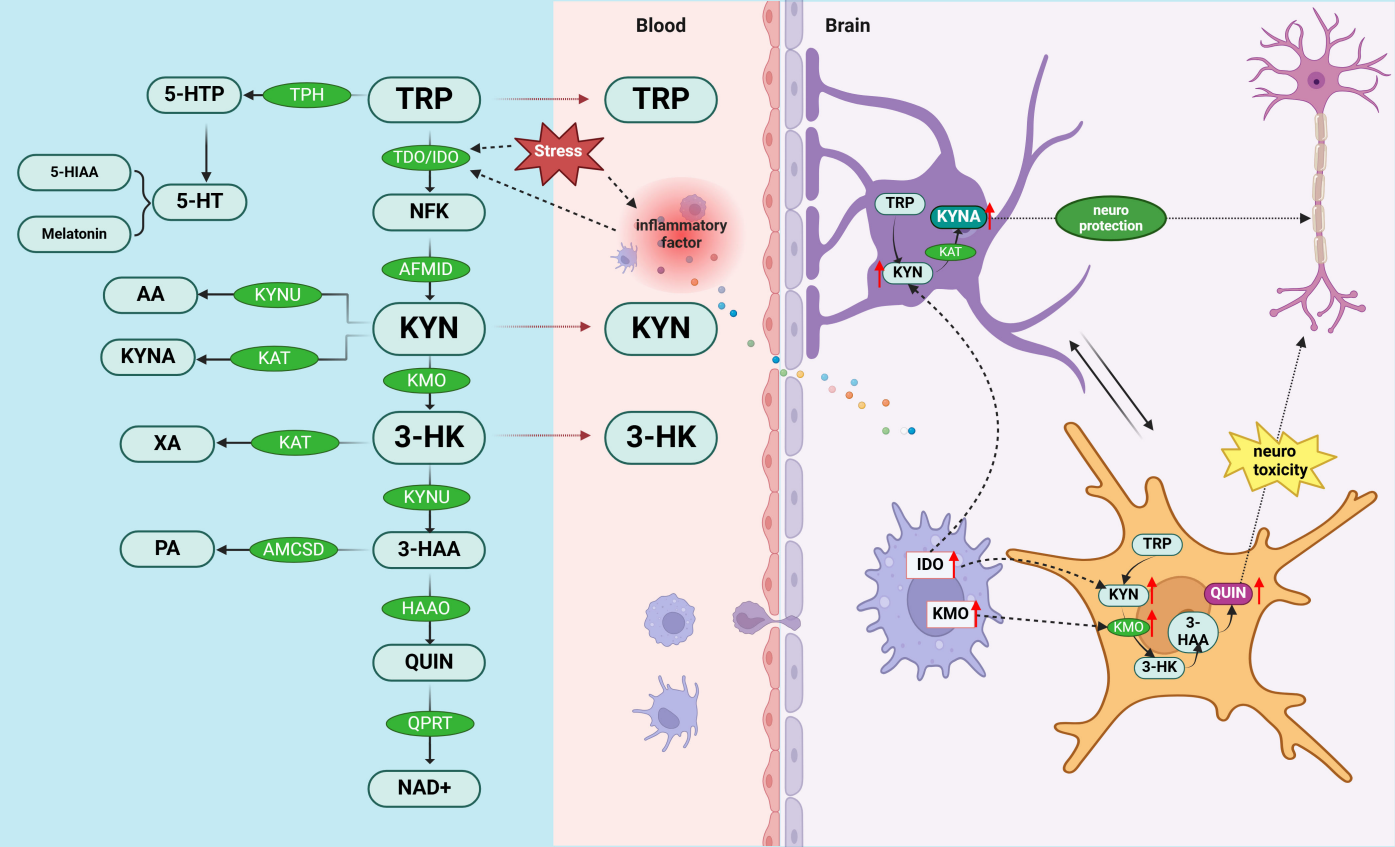
Potential mechanisms of PMS/PMDD pathogenesis involving 5-HT-KYN and inflammatory responses. The removal of tryptophan, the precursor of serotonin, from the diet induces premenstrual symptoms (86). TRY metabolism bifurcates into two distinct pathways: the “excitotoxic” and “neuroprotective” branches. Dysregulation in neurotoxic (such as 3-HK and QUIN) and neuroprotective (such as KYNA) TRYCATs is a key factor in the pathophysiology of mood disorders. These pathways are intricately linked to inflammatory responses: the excitotoxic pathway (TRY—KYN—3-HK—QUIN) is primarily metabolized by microglia, whereas the neuroprotective pathway (TRY—KYN—KYNA) is predominantly processed by astrocytes (99). 5-HTP, 5-hydroxytryptophan; 5-HT, serotonin; 5-HIAA, 5-hydroxyindole acetic acid Aminomuconic semialdehyde; AA, anthranilic acid; KYNU, kynureninase; KYNA, kynurenic acid; KAT, kynurenine aminotransferase; XA, xanthurenic acid; PA, positive affect; ACMSD, amino-B-carboxymuconate-semialdehyde-decarboxylase; TRP, tryptophan; TDO, tryptophan 2,3-dioxygenase; IDO, indole 2,3-dioxygenase; NFK, N’-formyl-L-kynurenine; AFMID, Arylformamidase; KYN, kynurenine; KMO, kynurenine mono-oxygenase; 3-HK, 3-hydroxy-kynurenine; 3-HAA, 3-hydroxy-anthranilic acid; HAAO, 3-hydroxyanthranilate-3,4-dioxygenase; QUIN, quinolinic acid; QPRT, quinolinate phosphoribosyl transferase; NAD+, nicotinamide adenine dinucleotide.

The shift from 5-HT synthesis to the KYN pathway in tryptophan (TRP) degradation is closely linked to inflammation, with the typically low baseline activity of IDO being upregulated by pro-inflammatory cytokines (26, 89). Clinical and preclinical studies provide robust evidence that inflammation can activate the KYN pathway (90). For instance, reduced levels of excitotoxic and/or neuroprotective metabolites have been observed in plasma or cerebrospinal fluid samples from individuals with major depressive disorder (MDD) (91) and those who have attempted suicide (92, 93). Furthermore, research indicates that patients treated with IFN-α exhibit an increased KYN/TRP ratio in their plasma and cerebrospinal fluid—an indicator of KYN pathway activation—correlated with the severity of depressive symptoms (94). In patients with PMS, there is a significant elevation in IgA responses to tryptophan catabolites (TRYCATs), particularly kynurenic acid, ferulic acid, quinolinic acid, and 3-hydroxykynurenine (95). Recent research underscores the pivotal role of imbalances in TRYCATs—encompassing neurotoxic compounds like 3-HK and QUIN, as well as neuroprotective ones like KYNA—in the pathophysiology of mood disorders (96–98). Moreover, the activation of the mucosal TRYCATs pathway is strongly associated with PMS, correlating with the activation of IDO in mucosal tissues (90, 95). The relationship between 5-HT-KYN pathway and inflammation is shown in **Figure 2**.

Based on the above studies, we hypothesize that increased inflammation in the brain may affect the 5-HT-KYN pathway which may result in the development of PMS/PMDD. This shift may be characterized by an imbalance between neurotoxic and neuroprotective metabolites due to an increase in the activity of pro-inflammatory cytokine up-regulated enzymes (IDOs) (95). Activation of the TRYCATs pathway (90), particularly in mucosal tissues, further supports the notion that inflammation is a central participant in PMS/PMDD, and that management of inflammation may be critical for symptom relief (96). Thus, inflammation-induced changes in the 5-HT-KYN pathway may be a key mechanism for mood disorders in PMS/PMDD.

### 4.3 Interaction mechanisms between the GABAergic system and inflammation

#### 4.3.1 GABA as a key mechanism in PMDD pathogenesis

Magnetic resonance spectroscopy studies reveal that while GABA levels decrease from the follicular to the luteal phase in healthy individuals, they increase in patients with PMDD (100, 101). Plasma GABA measurements indicate that women with a history of premenstrual irritability and severe depression exhibit lower GABA levels throughout both menstrual cycle phases (102). The GABA A receptor (GABAA_R), a transmembrane protein complex, modulates neuronal excitability by controlling chloride ion flow upon binding to GABA or an agonist, leading to neuronal hyperpolarization. Modulating the expression of receptors containing α4, α6, and δ subunits has been identified as an effective strategy for reducing neuronal excitability (103). It is suggested that increased GABAA_R subunit expression and decreased receptor sensitivity (lower affinity and reduced plasticity) result in diminished chloride influx, inhibiting GABA release from GABAergic interneurons, thereby reducing the inhibition of pyramidal neurons, increasing their excitability, and contributing to PMDD onset. Other viewpoints propose that neuroinflammation mediated by the GABAergic system plays a pivotal role in PMDD etiology, with Allopregnanolone (ALLO)-mediated GABAA_R activity being a significant pathogenic factor in PMDD (79, 104).

#### 4.3.2 Inflammatory mediators modulate GABAA_R transmission

GABAA_R agonists like topiramate and thymol have been shown to reduce the production of pro-inflammatory cytokines such as TNF-α, IL-17, IL-6, and the anti-inflammatory cytokine IL-10 in spleen cells (27). Research indicates that inflammatory mediators can influence the function and trafficking of GABAA_R. For instance, IL-6 has been observed to reduce GABAergic currents in the rat cortex (105) and to increase the expression of GABAA_R α5 subunit mRNA in the offspring’s hippocampus, which is associated with elevated apoptosis marker caspase-3 levels (106). A study involving the injection of GABA receptor agonists and antagonists in mice subjected to restraint stress demonstrated that central GABAA and GABAB receptors are involved in the suppressive regulation of plasma IL-6 levels in mice (107). TNF-α can also induce the internalization of GABAA_R in the hippocampus of both mice and rats (108, 109), leading to a reduction in inhibitory postsynaptic currents. Furthermore, high doses of TNF-α injected into the ventral tegmental area of rats have been shown to upregulate GABAA_R expression (110).

Data suggest that Lipopolysaccharide (LPS) activation of TLR4 reduces GABAergic synaptic activity primarily by decreasing GABA synthesis at presynaptic sites (111). LPS induces IL-1β release from microglia, which subsequently inhibits postsynaptic GABAA_R activity through protein kinase C (PKC) activation (112). Activation of GABAA_R α2 subunits in neurons triggers TLR4 signaling, leading to the activation of cAMP response element-binding protein (CREB) without engaging NF-κB transcription factor activation (phosphorylation/nuclear translocation), resulting in upregulation of corticotropin-releasing factor (CRF) and tyrosine hydroxylase (TH) (113). Similarly, exposure to the TLR3 viral mimic polyI has been found to increase immunoreactivity of the GABAA R α2 subunit protein in adult mice (114), along with an increase in GABAA R α3 subunit expression and a decrease in α4 and α5 subunit expression in the prefrontal cortex (115). Furthermore, downregulation of the GABAA_R α6 subunit using siRNA has been shown to exacerbate inflammatory pain in the temporomandibular joint of rats (116). Similarly, inhibition of GABAA_Rs has been reported to enhance inflammatory signaling pathways; for instance, blocking GABA_ARs with microtoxins increases NF-κB nuclear translocation in hippocampal slices (117).

**Figure 3:**
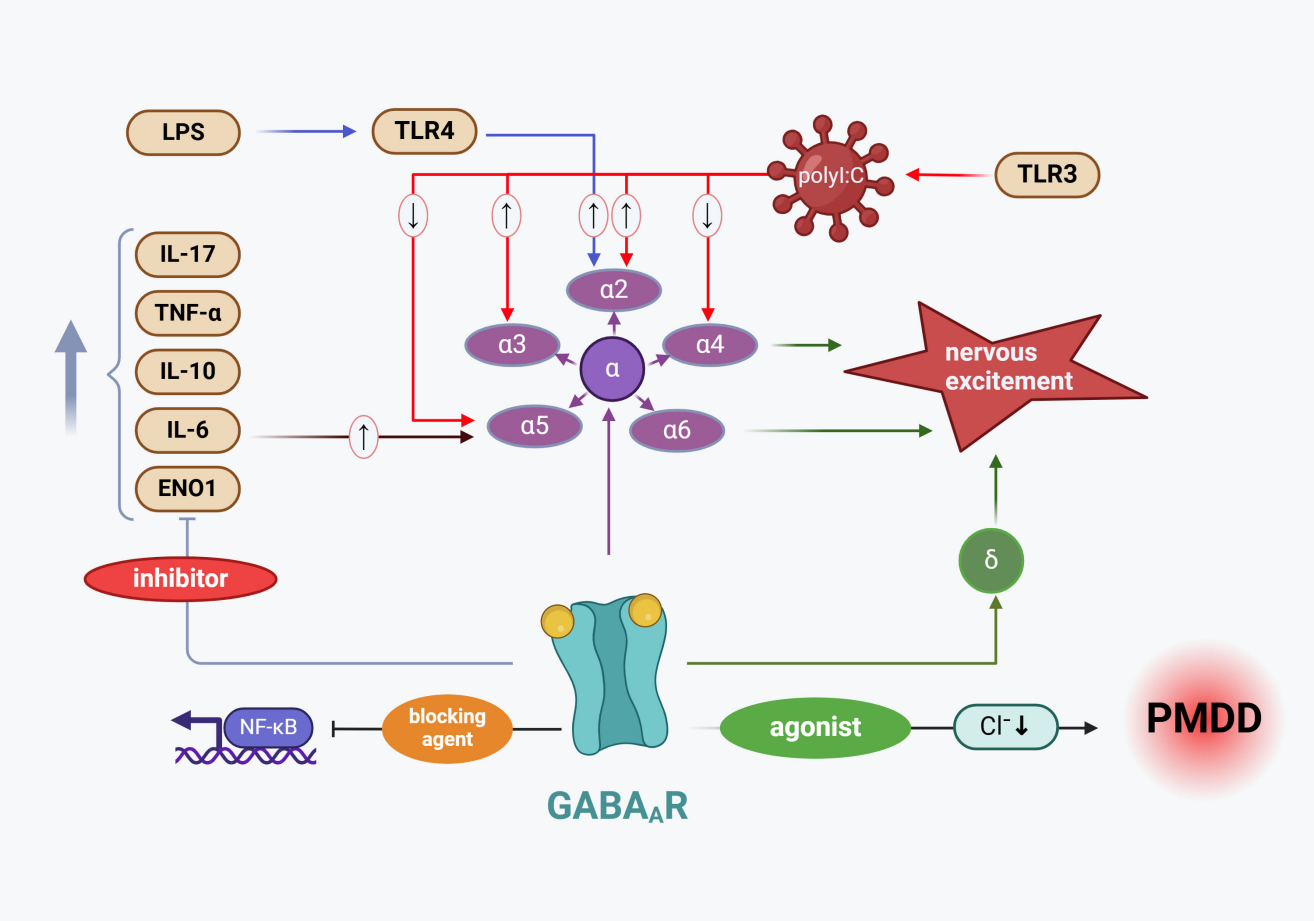
Mechanism by which inflammatory mediators regulate GABAA_R transmission leading to PMDD pathogenesis. The increased expression of GABAA_R subunits results in diminished chloride influx, which subsequently inhibits GABA release from GABAergic interneurons, contributing to PMDD development (104). Among the GABAA_R α subunits closely associated with PMDD, polyI (a TLR3 agonist) has been shown to elevate the expression of the α2 and α3 subunit proteins while reducing the expression of the α4 and α6 subunit proteins. Additionally, the LPS receptor TLR4 enhances the expression of the GABAA_R α2 subunit. Conversely, GABAA_R activity suppresses the expression of inflammatory cytokines, including TNF-α, IL-17, IL-6, and IL-10. LPS, Lipopolysaccharide; TLR4, Toll-like receptor 4; IL-17, interleukin-17; TNF-α, tumor necrosis factor alpha; IL-10, interleukin-10; IL-6, interleukin-6; ENO1, enolase 1 Gene; NF-κB, nuclear factor kappa-B; TLR3, Toll-like receptor 3; GABAA_R α2, Gamma-aminobutyric acid type A receptor subunit alpha 2; GABAA_R α3, Gamma-aminobutyric acid type A receptor subunit alpha 3; GABAA_R α4, Gamma-aminobutyric acid type A receptor subunit alpha 4; GABAA_R α5, Gamma-aminobutyric acid type A receptor subunit alpha 5; GABAA_R α6, Gamma-aminobutyric acid type A receptor subunit alpha 6; GABAAR δ, Gamma-aminobutyric acid type A receptor subunit delta.

In summary, inflammation plays a key role in regulating GABAergic activity, and inflammatory cytokines interfere with the function and trafficking of GABAA receptors (27). These cytokines reduce GABAergic currents, impair postsynaptic receptor activity, and alter the expression of GABAA receptor subunits, thereby exacerbating neuronal excitability (105, 118). Based on the above studies, we hypothesize that abnormal expression and sensitivity changes in GABAA receptors induced by inflammatory cytokines are one of the major pathological factors underlying the development of PMS/PMDD. The mechanism by which inflammatory mediators regulate GABAA receptor transmission and contribute to PMDD pathogenesis is illustrated in **Figure 3**.

### 4.4 BDNF and neuroimmune mechanisms

#### 4.4.1 Stress-induced changes in BDNF in patients with PMDD

Individuals with PMDD experience pronounced stress, which amplifies their negative emotional responses to stressors (119). This intensified stress response is associated with alterations in cortisol levels, particularly during the luteal phase, where cortisol secretion may increase in response to acute stress (30). Interestingly, during the Trier Social Stress Test (TSST), women with PMDD exhibit a blunted cortisol response (120). BDNF plays a key role in modulating cortisol responses to stress and has been shown to counteract the pathological effects of stress (121, 122). Notably, patients with PMDD display elevated compensatory BDNF levels during the luteal phase (123). Neurotrophic factors, including BDNF, are essential for neuronal survival, synaptic signaling, and integration, with BDNF being particularly important due to its role in brain plasticity. In adulthood, BDNF supports synaptic plasticity, learning, memory, hippocampal neurogenesis, and recovery following injury (124–127). Comasco et al. found that patients with PMDD with the BDNF Val66Met genotype exhibit decreased activation in the anterior cingulate cortex during the luteal phase compared to healthy women carrying the Met allele (128). Moreover, BDNF levels in patients with PMDD are significantly higher during the luteal phase than in the follicular phase, and this increase is strongly correlated with depressive symptoms (123, 129).

#### 4.4.2 Interactions between BDNF and inflammation

**Figure 4:**
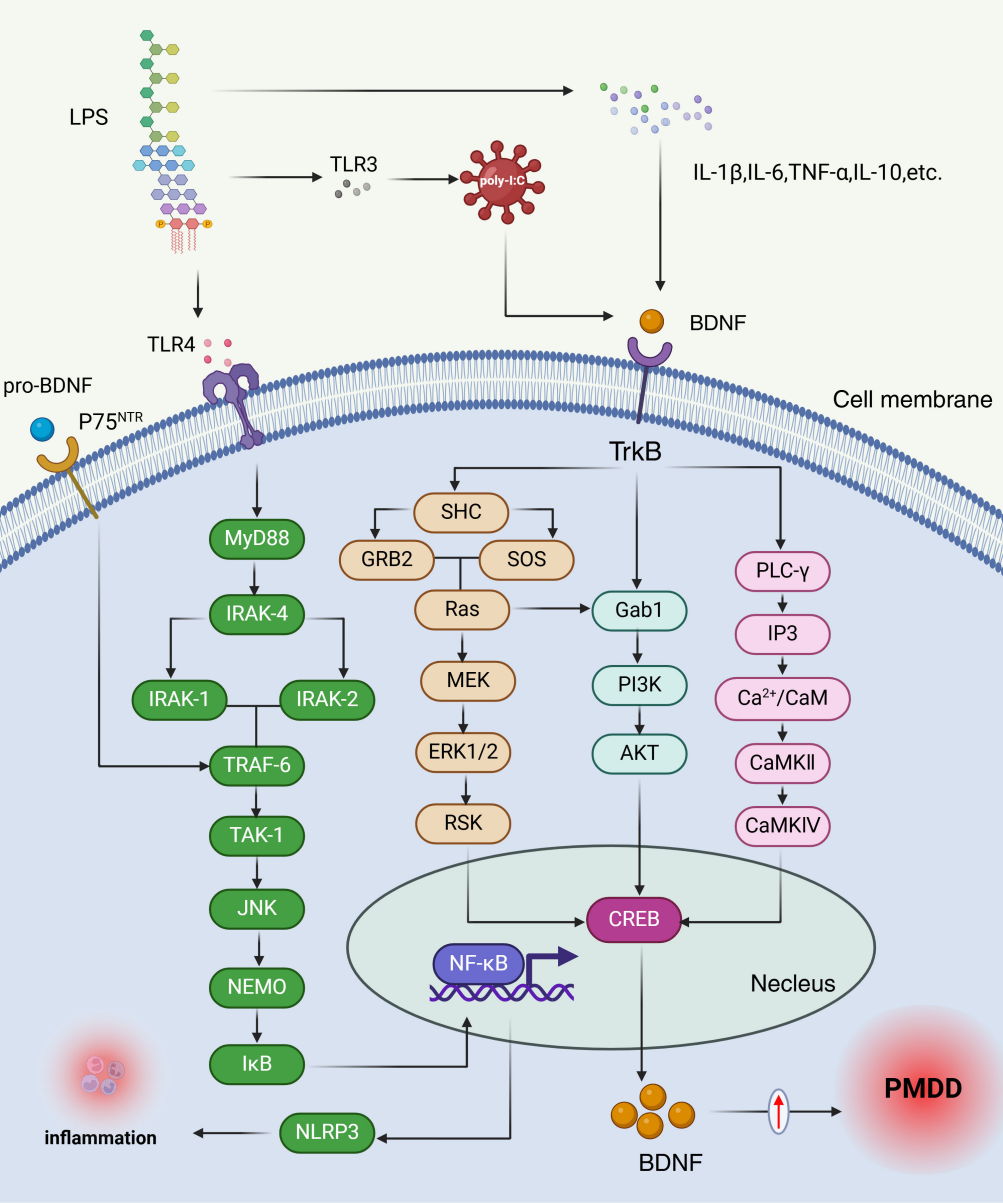
PMDD and stress-induced changes in BDNF and inflammation mechanisms. The elevated BDNF levels observed in the luteal phase of PMDD may reflect a compensatory response to chronic stress and increased inflammatory activity. LPS-induced inflammatory cytokines, including IL-1β, IL-6, TNF-α, and IL-10, inhibit BDNF expression and receptor phosphorylation, thereby disrupting BDNF signaling. When BDNF binds to its receptor TrkB, it triggers receptor activation, leading to dimerization and autophosphorylation of tyrosine residues within the cytoplasmic kinase domain. Each phosphorylation event initiates distinct signaling pathways: Ras/PI3K/Akt, Ras/MEK/Erk, or PLCγ, which in turn activate transcription factors such as CREB and NF-κB. Additionally, proBDNF interacts with the P75 neurotrophin receptor (P75^NTR^), initiating the IRAK/TRAF6/JNK cascade, which also activates NF-κB. NF-κB is a key regulator of cellular stress responses and inflammatory processes (147). LPS, Lipopolysaccharide; TLR4, Toll-like receptor 4; TLR3, Toll-like receptor 3; p75NTR, Neurotrophin Receptor P75; MyD88, Myeloid Differentiation Primary Response Protein 88; IRAK-4, Interleukin-1 Receptor-Associated Kinase 4; IRAK-1, Interleukin-1 Receptor-Associated Kinase 1; IRAK-2, Interleukin-1 Receptor-Associated Kinase 2; TRAF-6, TNF receptor associated factor 6; TAK-1, transforming growth factor beta-activated kinase 1; JNK, c-Jun N-terminal kinase; NEMO, NF-κB Essential Modulator; IκB, inhibitor of NF-κB; SHC, SHC transforming protein; BDNF, brain-derived neurotrophic factor; Trkb, Tropomyosin receptor kinase B; GRB2, growth factor receptor-bound protein 2; SOS, son of sevenless homolog; Ras, rat sarcoma; MEK, mitogen-activated protein; ERK1/2, extracellular-signa1regulated kinase; RSK, ribosomal S6 kinase; NF-κB, nuclear factor kappa-B; Gab1, GRB2-Associated Binding Protein 1; PI3K, Phosphoinositide 3-Kinase; AKT, protein kinase B; PLC-γ, Phospholipase C gamma 1; CREB, CAMP-response element binding protein; IP3, inositol triphosphate; Ca2+/CaM, Ca/calmodulin-dependent protein kinases; CaMKII, Calcium Calmodulin Dependent Protein Kinase II; CaMKIV, Calcium-/Calmodulin-Dependent Protein Kinase IV.

BDNF is a widely studied neurotrophic factor that promotes the proliferation of neurons and glial cells through various molecular mechanisms during neuroinflammatory processes (130). Research indicates that microglia are essential for establishing proper synaptic connections during development and maturation, a process often mediated by BDNF (131, 132). Additionally, in models of astrocyte damage induced by LPS, elevated levels of IL-6, IL-1β, and BDNF have been observed in LPS-stimulated normal human astrocytes (NHA). In turn, BDNF can regulate the survival and proliferation of LPS-induced normal human astrocytes (NHA) via the PI3K/AKT signaling pathway (133). Inflammatory cytokines have consistently been shown to affect neuronal development and apoptosis (134, 135). Stress and its associated inflammatory cytokines can negatively impact neurogenesis and neural plasticity (136, 137). Several studies have explored the effects of inflammation on BDNF expression in the brain. For instance, the administration of pro-inflammatory cytokines or LPS significantly reduces BDNF levels. Specifically, LPS injections lead to a significant decrease in mature BDNF levels in both the hippocampus and cortex, while IFN-α treatment reduces systemic BDNF levels (138, 139). Other neurotrophic factors, such as nerve growth factor (NGF) and neurotrophin-3 (NT-3), also exhibit varying degrees of reduction (140). Substantial evidence suggests that inflammation impairs BDNF/TrkB expression, with inflammatory cytokines affecting the phosphorylation of the BDNF receptor TrkB, thereby disrupting BDNF signaling (141). Gibney et al. demonstrated that polyI administration increases the expression of inflammatory cytokines, triggering an inflammatory response while concurrently downregulating BDNF and TrkB expression in the hippocampus and cortex. This downregulation may be linked to behavioral issues such as depression and anxiety (142). PMDD and stress-induced changes in BDNF and inflammation Mechanisms are shown in **Figure 4**.

In summary, The elevated BDNF levels observed in the luteal phase of PMDD may reflect a compensatory response to chronic stress and increased inflammatory activity, However, this increase in BDNF is often maladaptive, leading to disruptions in synaptic plasticity and neuronal survival (143, 144), which may underlie the mood disturbances seen in PMDD. In future studies, the latest research can be integrated to observe changes in BDNF levels in PMS/PMDD through methods such as cognitive therapy, physical therapy (e.g., environmental enrichment) (145), or nutritional supplements (N-acetylcysteine) (146), in order to explore effective ways to prevent PMS/PMDD. 

### 4.5 Biological mechanisms of the HPA axis

#### 4.5.1 HPA axis dysfunction in PMDD

The HPA axis, as a central stress response system, orchestrates the adrenal release of hormones in response to both external and internal stressors. Upon the release of glucocorticoids (GCs), these hormones exert wide-ranging effects throughout the body, modulating various physiological processes *via* genomic and non-genomic mechanisms mediated by glucocorticoid receptor (GR) (148). These processes encompass the regulation of metabolism, cell growth, inflammation, immune responses, development, and reproduction (149, 150). Consequently, the HPA axis is pivotal in managing stress responses and maintaining homeostasis. Beyond their physiological functions, GCs provide negative feedback to the HPA axis through multiple mechanisms. They influence the axis components directly and modulate brain structures such as the hippocampus, prefrontal cortex, and amygdala indirectly (151).

A study investigated the effects of PMS and the menstrual cycle on the HPA axis, the SNS axis, and psychological responses to the TSST, findings revealed that women with PMS exhibited a blunted cortisol response to the TSST compared to controls. Moreover, diminished HPA axis reactivity in PMS appears to correlate with the severity of PMS symptoms (28). Heart rate variability (HRV), total power, and high-frequency power—indicators of overall autonomic and parasympathetic nervous system activity—were significantly lower during the luteal phase in women with PMS compared to the follicular phase. In individuals with severe PMDD, heart rate variability and power spectral components were markedly reduced across all menstrual cycle phases compared to other groups (152). Furthermore, women with PMDD demonstrated lower stroke volume, cardiac output, and cortisol levels both at rest and under stress, while norepinephrine levels and total peripheral resistance were significantly elevated compared to controls (153). Given that PMDD is associated with increased daily stress (153, 154) and is exacerbated by stressful life events (155, 156), the dysfunction in stress responses is believed to be linked to these conditions. In conclusion, the evidence suggests that women with PMDD exhibit lower activation of both the HPA and SNS axes compared to those without PMDD (119).

#### 4.5.2 Interactions between the HPA axis and the immune system

The HPA axis, inflammation, and the immune system interact in intricate and complex ways. While the HPA axis and GCs are well-established as regulators of immune responses, the immune system can reciprocally influence HPA axis function. Pro-inflammatory cytokines, in particular, are known to activate the HPA axis and stimulate glucocorticoid release (25). During neuroinflammation, these cytokines can directly trigger HPA axis activation by affecting hypothalamic cells. Additionally, peripheral inflammatory mediators can cross the BBB, either through alterations in endothelial cells of adjacent organs or by modifying BBB permeability (157). These mediators can also communicate peripheral inflammation to the central nervous system *via* vagal afferent fibers (158). Furthermore, peripheral inflammation can prompt the release of signaling molecules in the brain, such as myeloid differentiation primary response gene 88 (MyD88), cyclooxygenase-1 (COX-1), COX-2, and prostaglandin E2, which subsequently stimulate the HPA axis (159, 160). In conclusion, the HPA axis leverages the diverse actions of GCs to address potential disruptions to homeostasis. Among these actions, the immunomodulatory effects of GCs are critical for maintaining equilibrium, particularly under conditions of high stress. The role of GCs in immune regulation is extensive and intricate, influencing nearly every aspect of immune function (157).

To sum up, The pathogenesis of PMS/PMDD appears to be linked to dysregulation in the HPA axis and its interactions with the immune system. Women with PMS/PMDD exhibit impaired HPA axis function, including blunted cortisol responses to stress and altered autonomic nervous system activity, suggesting a diminished ability to cope with stress (119). This dysfunction could be compounded by inflammatory processes, as the immune system can influence HPA axis activity, exacerbating stress responses (25). Pro-inflammatory cytokines, released during periods of neuroinflammation, may further trigger HPA axis activation, creating a feedback loop that impairs both emotional regulation and immune function (157). Given the complex interplay between stress, immune responses, and hormonal regulation, HPA axis dysfunction may contribute significantly to the pathophysiology of PMS/PMDD, where both stress reactivity and immune system alterations synergistically worsen the disorder’s symptoms.

## 5 Conclusions

This review elucidates the intricate neurobiological mechanisms underlying the pathophysiology of premenstrual syndrome (PMS) and premenstrual dysphoric disorder (PMDD), with a particular focus on the roles of neuroinflammation and stress. Emerging evidence underscores the bidirectional relationship between chronic stress and inflammation, highlighting how these factors converge to exacerbate the central nervous system (CNS) dysfunction observed in PMS/PMDD. Stressors, particularly those related to hormonal fluctuations, initiate a cascade of inflammatory responses that impact brain regions critical to mood regulation, these inflammatory processes are closely linked to alterations in neurotransmitter systems, including 5-HT-KYN pathway, GABAergic system, HPA axis, BDNF, and HPO axis, which play pivotal roles in mood stability and emotional regulation. In this study, research using PMS/PMDD as an animal model was conducted primarily with female rodents. However, for studies on inflammation, 5-HT, GABA, BDNF, HPA axis, and other aspects, we have made efforts to reference research conducted on female rodents. Nevertheless, some studies included either both sexes of rodents or male animals, which is a limitation of our study. However, for the sake of completeness in the argument, we had to reference these studies. Together, stress-induced neuroinflammation constitutes a key pathogenic mechanism in the development and exacerbation of PMS/PMDD, offering potential therapeutic targets for future interventions aimed at modulating both inflammatory and stress response pathways. Although research on the link between PMDD and inflammation remains limited, this connection represents a promising frontier in the field, warranting heightened focus from researchers in the years ahead.

## Glossary

3-HAA: 3-hydroxy-anthranilic acid
3-HK: 3-hydroxy-kynurenine
5-HIAA: 5-hydroxyindole acetic acid,Aminomuconic semialdehyde
5-HT: serotonin
5-HTP: 5-hydroxytryptophan
AA: anthranilic acid
ACC: anterior cingulate cortex
ACMSD: amino-B-carboxymuconatesemialdehyde-decarboxylase
ACOG: American College of Obstetricians and Gynecologists
ACTH: adrenocorticotropic hormone
ADIOL: 5-Androstene-3b,17b-diol
AFMID: Arylformamidase
AKT: protein kinase B
ALLO: allopregnanolone
ANS: autonomic nervous system
AP-1: activated protein-1
APA: American Psychological Association
ATP: adenosine triphosphate
AVP: arginine vasopressin
BBB: blood-brain barrier
BDNF: brain-derived neurotrophic factor
Ca^2+^/CaM: Ca/calmodulin-dependent protein kinases
CaM: calmodulin
CaMKII: calcium/calmodulin-dependent protein kinase II
CaMKIV: calcium/calmodulin-dependent protein kinase IV
CCL2: chemokine (C-C motif) ligand 2
COX: cyclooxygenase
CREB: CAMP-response element binding protein
CRH: corticotropin-releasing hormone
CRP: C-reactive protein
CtBP: C-terminal binding protein
DAMPs: damage-associated molecular patterns
DHEA: dehydroepiandrosterone
DRSP: Daily Record of Severity of Problems
DSM-5: Diagnostic and Statistical Manual of Mental Disorders, Fifth Edition
DSM-5-TR: Diagnostic and Statistical Manual of Mental Disorders, Fifth Edition, Text Revision
eNOS: endothelial nitric oxide synthase
ERK1/2: extracellular-signa1regulated kinase
ERα: estrogen receptors α
ERβ: estrogen receptors β
Gab1: GRB2-Associated Binding Protein 1
GABA: γ-aminobutyric acid
GABAA_R: GABA A receptor
GCs: glucocorticoids
GILZ: glucocorticoid-induced leucine zipper
GM: gray matter
GR: glucocorticoid receptor
GRB2: growth factor receptor-bound protein 2
GREs: glucocorticoid response elements
GSK-3β: recombinant glycogen synthase kinase 3β
HAAO: 3-hydroxyanthranilate-3,4-dioxygenase
HPA: hypothalamic-pituitary-adrenal
HPO: hypothalamic-pituitary-ovary
HRV: heart rate variability
hs-CRP: hypersensitive C-reactive protein
HSD17B14: 17b-hydroxysteroid dehydrogenase type 14
hsp70: heat shock protein 70
hsp90: heat shock protein 90
ICAM-1: intercellular adhesion molecule 1
IDO: indole 2,3-dioxygenase
IFN-γ: interferon gamma
IgA: immunoglobulin A
IL-10: interleukin-10
IL-12: interleukin-12
IL-17: interleukin-17
IL-1β: interleukin-1 Beta
IL-6: interleukin-6
IL-9: interleukin 9
iNOS: inducible nitric oxide synthase
IP3: inositol trisphosphate
IRAK-1: Interleukin-1 Receptor-Associated Kinase 1
IRAK-2: Interleukin-1 Receptor-Associated Kinase 2
IRAK-4: Interleukin-1 Receptor-Associated Kinase 4
IκB: inhibitor of NF-κB
NK: c-Jun N-terminal kinase
KAT: kynurenine aminotransferase
KMO: kynurenine mono-oxygenase
KYN: kynurenine
KYNA: kynurenine acid
KYNU: kynureninase
LC: locus coeruleus
LPS: lipopolysaccharide
MDD: major depressive disorder
MEK: mitogen-activated protein
MKP-1: mitogen-activated protein kinase phosphatase-1
MMP-9: matrix metalloproteinase 9
MMPs: matrix Metalloproteinases
mPFC: medial prefrontal cortex
MSK1: mitogen- and stress-activated protein kinase 1
mTOR: mammalian target of rapamycin
MyD88: Myeloid Differentiation Primary Response Protein 88
NA: negative affect
NAD+: micotinamide adenine dinucleotide
NEMO: NF-κB Essential Modulator
NFK: N’-formyl-L-kynurenine
NGF: nerve growth factor
NHA: normal human astrocytes
NK: natural killer
NLRP3: NOD-like receptor family pyrin domain containing 3
NO: nitric oxide
NOD: nucleotide-binding oligomerization domain
NT-3: neurotrophin-3
PA: positive affect
PGE2: prostaglandin E2
P-gp: P-glycoprotein (or P-glycoprotein 1)
PI3K: Phosphoinositide 3-Kinase
PLC-γ: Phospholipase C gamma 1
PMDD: premenstrual dysphoric disorder
PMS: premenstrual syndrome
PNS: parasympathetic nervous system
POMC: pro-opiomelanocortin
PP5: protein phosphatase 5
PVN: hypothalamic paraventricular nucleus
QPRT: quinolinate phosphoribosyl transferase
QUIN: quinolinic Acid
Ras: rat sarcoma
ROS: reactive oxygen species
RSK: ribosomal S6 kinase
SAM: sympathetic-adrenal-medullary
SHC: SHC transforming protein
SNS: sympathetic nervous system
SOS: son of sevenless homolog
SSRIs: selective serotonin reuptake inhibitors(s)
STAT3: signal transducer and activator of transcription 3
TAK-1: transforming growth factor beta-activated kinase 1
TDO: tryptohan 2,3-dioxygenase
TJs: tight junctions
TLR4: Toll-like receptor 4
TNF-α: tumor necrosis factor alpha
TPH: tryptophan hydroxylase
TRAF-6: TNF receptor associated factor 6
Trkb: Tropomyosin receptor kinase B
TRP: tryptophan
TRYCAT: tryptophan catabolites
TSST: trier social stress test
VCAM-1: vascular cell adhesion molecule 1
VEGF-A: vascular endothelial growth factor
XA: xanthurenic acid
α-MSH: α-melanocyte-stimulating hormone
β-EP: β-endorphin
